# Localization and diagnosis framework for pediatric cataracts based on slit-lamp images using deep features of a convolutional neural network

**DOI:** 10.1371/journal.pone.0168606

**Published:** 2017-03-17

**Authors:** Xiyang Liu, Jiewei Jiang, Kai Zhang, Erping Long, Jiangtao Cui, Mingmin Zhu, Yingying An, Jia Zhang, Zhenzhen Liu, Zhuoling Lin, Xiaoyan Li, Jingjing Chen, Qianzhong Cao, Jing Li, Xiaohang Wu, Dongni Wang, Haotian Lin

**Affiliations:** 1 School of Computer Science and Technology, Xidian University, Xi’an, China; 2 School of Software, Xidian University, Xi’an, China; 3 State Key Laboratory of Ophthalmology, Zhongshan Ophthalmic Center, Sun Yat-sen University, Guangzhou, China; 4 School of Mathematics and Statistics, Xidian University, Xi’an, China; Soochow University Medical College, CHINA

## Abstract

Slit-lamp images play an essential role for diagnosis of pediatric cataracts. We present a computer vision-based framework for the automatic localization and diagnosis of slit-lamp images by identifying the lens region of interest (ROI) and employing a deep learning convolutional neural network (CNN). First, three grading degrees for slit-lamp images are proposed in conjunction with three leading ophthalmologists. The lens ROI is located in an automated manner in the original image using two successive applications of Candy detection and the Hough transform, which are cropped, resized to a fixed size and used to form pediatric cataract datasets. These datasets are fed into the CNN to extract high-level features and implement automatic classification and grading. To demonstrate the performance and effectiveness of the deep features extracted in the CNN, we investigate the features combined with support vector machine (SVM) and softmax classifier and compare these with the traditional representative methods. The qualitative and quantitative experimental results demonstrate that our proposed method offers exceptional mean accuracy, sensitivity and specificity: classification (97.07%, 97.28%, and 96.83%) and a three-degree grading area (89.02%, 86.63%, and 90.75%), density (92.68%, 91.05%, and 93.94%) and location (89.28%, 82.70%, and 93.08%). Finally, we developed and deployed a potential automatic diagnostic software for ophthalmologists and patients in clinical applications to implement the validated model.

## Introduction

Pediatric cataract is a common ophthalmic disease seriously causing permanent visual impairment and thus dramatically reducing the quality of life [1]. A world health report [2] indicates that pediatric cataract is one of the major causes of childhood blindness; it affects approximately 200,000 children worldwide, with an estimated prevalence of 4.24 per 10,000 live births [3]. The asymptomatic progression of pediatric cataracts patients is hard to be realized and detected at early age, which is difficult for their parents to identify as well [4]. Once pediatric cataracts enter a more severe stage, current intervention procedures are no longer available to prevent vision impairment [5]. Therefore, it is critical to diagnose pediatric cataracts with high accuracy at early stage, which can help ophthalmologists arrange appropriate and timely treatment to prevent disease progression.

In clinical practice, comprehensive evaluation of pediatric cataracts is often manually assigned by well-experienced ophthalmologists to each slit-lamp image [6, 7]. However, this manual diagnosis scheme is not only a waste of resource of excellent ophthalmologists, but also is subjective and time-consuming. In recent decades, combined with slit-lamp images and other ocular images, computer aided diagnosis (CAD) methods have become the dominant alternatives for controlling ophthalmic diseases and early treatment and have been initially investigated by scientists, ophthalmologists, and computer vision researchers [8]. A ranking method based on slit-lamp images proposed by Wei Huang [9] achieved an acceptable grading for nuclear cataracts. The senile cataracts classification and grading system based on fundus images was presented in [10], which extracted local features using the wavelet transformation and sketch and provided a possible method to reduce the burden of experienced ophthalmologists. Shaohua Fan et al. proposed an automatic classification method for nuclear sclerosis from slit-lamp images using linear regression [11]. Huiqi Li et al. extracted local features from slit-lamp images and considered the nuclear cataract grading task as a support vector regression [12]. In addition, there are still some reasonable CAD methods based on other ocular images achieving effective results [13–15].

**Fig 1:**
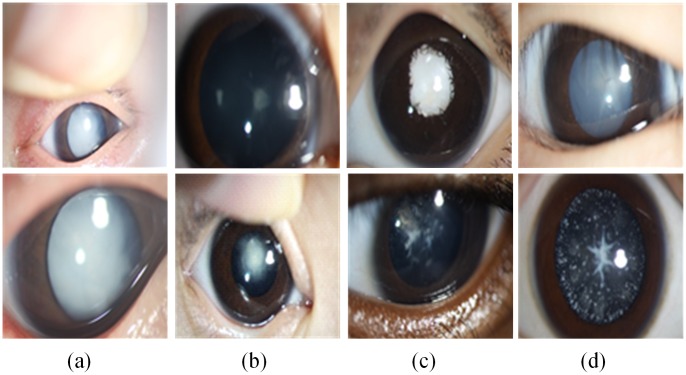
Examples of various challenges associated with complex slit-lamp images. **(a)** The different amplification factors of optical device. **(b)** The non-cooperation of patient and the different angle of the photographer. **(c)** The complications of pediatric cataract. **(d)** The noise of eyelashes and white highlights.

However, relative to senior cataracts and other ophthalmic diseases, the phenotypes of pediatric cataracts are varied and abundant. The slit-lamp images for pediatric cataracts are complex and clinically challenging [1, 16, 17]. The aforementioned CAD methods can’t tackle such a difficult situation and be directly applied on pediatric cataracts. In our previous study, our team conducted a series of CAD approaches consisting of feature extraction and classification for pediatric cataracts and achieved encouraging results. However these conventional CAD methods are subject to low accuracy and cannot be implemented effectively in clinical applications. The complexity of pediatric cataract is manifested primarily as high noise levels and complex disease phenotypes shown in Fig 1. For example, the ratio of the lenses in the two slit-lamp images of column (a) significantly differs as a result of the amplification factors of the optical device. The slit-lamp images in column (b) are blurrier due to an uncooperative patient and the angle of the photographer. The images in column (c) differ from the remaining columns because patients have another ophthalmic disease and pediatric cataracts, and the large number of eyelashes produces additional noise in column (d). In addition, white highlights and finger reflections in the lens of almost all slit-lamp images occur from the reflection of the light source. Therefore, these factors pose significant challenges for computer-aided automated diagnosis of pediatric cataracts based on slit-lamp images.

Recently, deep learning convolutional neural network (CNN) methods have gained considerable popularity since they offer superior performance in the field of image recognition tasks [18–22]. The CNN is an end-to-end learning model that avoids image pre-processing, requires no expert knowledge and extracts relevant high-level features directly from the raw image. The CNN architecture is inspired by the visual cortex of cats in Hubel’s and Wiesel’s early work [23]. In particular, Krizhevsky [24] performed object classification and won first prize in the ImageNet Large Scale Visual Recognition Challenge 2012 using a deeper CNN. This was followed by the emergence of many improved algorithms and applications of CNN [25–27], and similar CNN architectures can be generalized to solve various image classification tasks. Based on these factors and research progress, we employ a deep learning CNN method to investigate slit-lamp images diagnosis in the context of pediatric cataracts. The superior performance indicates that it can provide an effective solution for clinical characterization of pediatric cataracts when related studies are scarce at present; and this approach will probably offer a useful reference for other ophthalmic diseases researchers. The main contribution of this study focuses on three aspects. 1) For the characteristics and clinical examination mode of slit-lamp images for pediatric cataracts, we propose three-degree grading for pediatric cataracts in terms of morphology and then present an automatic, objective and efficient localization and diagnosis method for slit-lamp images that achieves exceptional performance. 2) Detailed comparison experiments are conducted by exploiting slit-lamp images for pediatric cataracts classification and grading, where several conventional feature extraction methods and classifiers are used for comparison with the CNN method. During this process, we combine the SVM classifier with the deep features extracted from the CNN to further investigate and verify the effectiveness of deep features of CNN. 3) Finally, we develop and deploy a web-based CAD software for patients and ophthalmologists based on slit-lamp images to achieve effective clinical application.

## Method

### Slit lamp photography method

The slit-lamp-adapted anterior segmental photography (BX900, Haag-Streit AG, Köniz, Switzerland) was used throughout our study. And the pediatric cataract was assessed using the standard procedure of the operation manual [28]. Detailed protocol description: The slit lamp, a high-intensity light source instrument, is used to shine a thin sheet of light into the eye to examine the anterior segment and posterior segment of the human eye [6]. While a patient is seated in the examination chair, they rest their chin and forehead on a support to steady the head. Using the slit lamp, an ophthalmologist then proceeds to examine ocular tissues including the eyelid, sclera, conjunctiva, iris, natural crystalline lens, and cornea. If medial, especially that of the cornea, are opaque, optical section images are often impossible depending on the severity of ophthalmic diseases. In these cases, the direct diffuse illumination is an effective examination method. For this, we open the slit very widely, and then a diffuse, attenuated survey illumination is produced by inserting a ground glass screen or diffuser in the illuminating path. It is applied mainly to illuminate as much of the eye and its adnexa at once for general observation.

### Ethical approval

The slit-lamp images collection and label setting were implemented mainly by the ophthalmologists in Zhongshan Ophthalmic Center. The research protocol involving patients was approved by the Institutional Review Board/Ethics Committee of Xidian University and Zhongshan Ophthalmic Center of Sun Yat-sen University. Meanwhile, we also need to emphasize that all of the sensitive information of patients has been removed beforehand in this study. Therefore, we strictly ensure the personal information of patient is confidential and anonymous. For all involved patients, we have obtained the written informed consents from their parents according to Childhood Cataract Program of the Chinese Ministry of Health (CCPMOH) [29].

### Overall diagnosis framework for slit-lamp images

**Fig 2:**
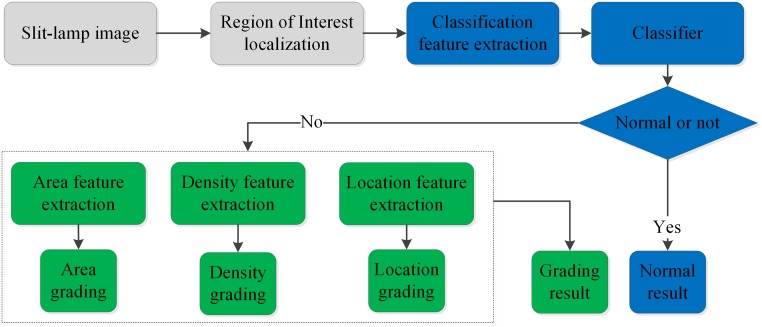
Flow chart of the automatic diagnosis process for slit-lamp images. The gray, blue and green block diagram represent the ROI of lens localization, classification and grading procedure respectively. (ROI: region of interest).

The proposed CAD framework for slit-lamp images is shown in Fig 2 and primarily consists of three parts: automatic localization for lens ROI, classification and three-degree grading. First, the lens ROI is localized accurately using twice-performed Candy detection and Hough transformation and is then fed into the CNN model to extract high-level features and implement classification and grading. If one sample is predicted to be normal, we record this determination in the formation of the normal subset. Otherwise, the remaining three classifiers are performed to determine its severity, thus forming another grading subset. Finally, these two results are sent to ophthalmologists to determine which therapeutic schedule will be adopted for the patient based on the severity of the slit-lamp image.

**Fig 3:**
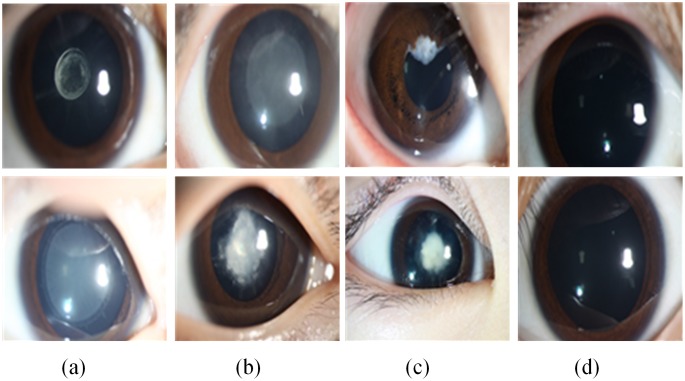
Examples of slit-lamp images of three-degree grading. From (a) to (c) are area, density, and location grading, in which the upper images represent mild disease and the lower images are severe. (d) The normal images for comparison.

For the three-degree grading, in general, the researcher and ophthalmologist divide the cataract into four grades [10, 12] (normal, mild, moderate and severe), but this grading is too vague and subjective for effective individualized treatment. By investigating the lens opacity and morphology, we proposed three grading degrees of the slit-lamp containing opacity area (limited and extensive), density (dense and transparent) and location (central and peripheral) in conjunction with three leading ophthalmologists to evaluate severity. In clinical classification, the slit-lamp image of which the cataract region is more than half of the pupil is defined as extensive, otherwise limited. If the cataract density of patient blocks the external light completely, this case is defined dense, otherwise transparent. If the visual axis of the pupil is covered completely by the pediatric cataract, this case is defined central, otherwise peripheral. The final therapeutic schedule was determined by the results of these three grading degrees. For each binary classification assessment, each slit-lamp image was assessed by three experienced pediatric ophthalmologists independently. And the agreement results were determined by these three ophthalmologists after discussion. As is shown in Fig 3, comparative samples of the three grading types—area, density, and location in columns (a) to (c) and two normal slit-lamp images in column (d)—are illustrated. The upper samples are mild, whereas the lower samples are severe. For example, column (a) contains two slit-lamp images with limited and extensive opacity. Generally, the extensiveness and density of the lens opacity is positively correlated with severity of the pediatric cataract. And opacity in the central part of lens indicates a serious condition.

### Lens localization based on the candy operator and hough transform

It is well recognized that slit-lamp images are complex and contain large amount of noise. Pediatric cataracts occur only in the lens rather than other regions such as eyelids and sclera. If the entire original slit-lamp image were analyzed using the deep learning model, a number of features irrelevant to pediatric cataracts would be extracted, seriously affecting the accuracy rates of classification and grading. We therefore obtain the region of interest (ROI) in the original slit-lamp image by careful cropping that maintains only the whole pupil and minimizes the noise around the pupil area. Moreover, the user-defined manual cropping of the lens area is so time-consuming and laboursome that an automatic localization method is urgently required.

We observed that the boundary between the iris and lens is approximately a circle in the original slit-lamp images and that the lens is surrounded by the iris. These characteristics provided a basis for lens and iris detection. Many studies have identified the ROI of the iris for personal identification using various computer vision methods [30–32]. In this paper, we investigated the relatively successful Candy operator and Hough transform approach [33, 34] and improved them to automatically localize the lens region in the original slit-lamp images, as is detailed in the figure below.

**Fig 4:**
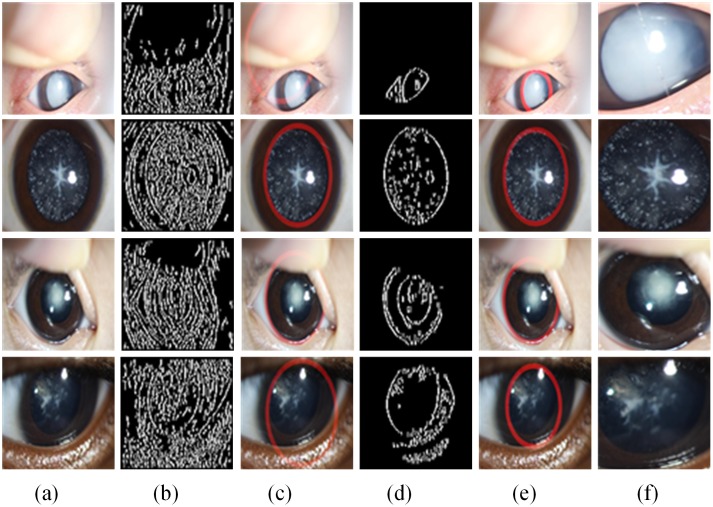
Examples of lens localization. **(a)** Representative original images from Fig 1. **(b)** Edge detection using the Candy operator on the H component of the hue, saturation, and value (HSV) color space. **(c)** The regions of lens localization using the Hough transform correspond to (b). **(d)** Edge detection using the S component. **(e)** Final lens localization. **(f)** Cropped images for constructing slit-lamp datasets.

As is shown in Fig 4, the original slit-lamp images (Fig 4(a)) are first converted into hue, saturation, and value (HSV) space from red, green, and blue (RGB), and the general contour of the original image (Fig 4(b)) is found using the Candy operator on the H component of HSV; then, the general region of lens can be identified as the red circular bounding boxes in (Fig 4(c)) using the Hough transform. Because of the diversity and complexity of the phenotypes and characteristics of slit-lamp images, the lens region could not be located accurately by any one component of the color space. After obtaining the approximate region of the lens through the above steps, we set all pixel values outside of the circle to be zero to eliminate most of the noise. Then, the S component of HSV that corresponds to the same located region of the H component is operated by the Candy operator and the Hough transform, which enables the contour to be detected, as is shown in (Fig 4(d)), and the lens region to be accurately identified, as is shown in (Fig 4(e)), which is followed by the resizing of all cropped regions to 256×256 pixels (Fig 4(f)) to assemble a pediatric cataract database.

### Deep convolutional neural network

**Fig 5:**
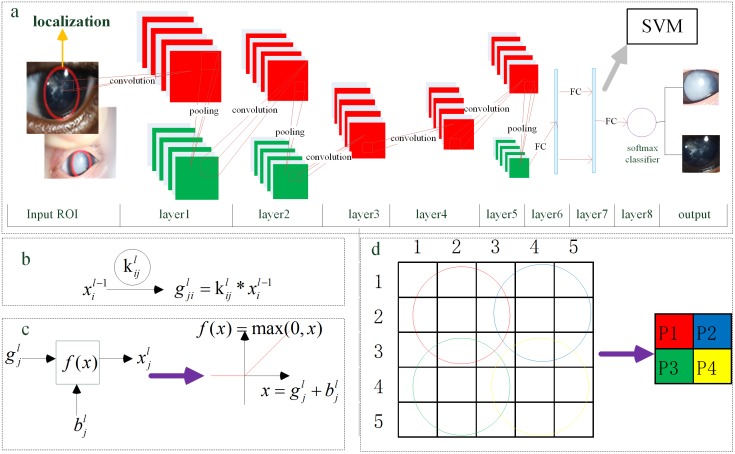
The architecture of the deep convolutional neural network. **(a)** Main layers and connections of the CNN that is employed in our study. It consists of five convolutional and overlapping max pooling layers, which are indicated by red rectangles and green rectangles respectively, followed by three fully-connected layers. **(b), (d)** Convolutional and overlapping max pooling operations are represented respectively. **(c)** The non-saturating activation function ReLU was represented. (ReLU: rectified linear units; CNN: convolutional neural network; SVM: support vector machine; ROI: region of interest; FC: full-connected operation; P1~P4: pixel value after pooling operation).

The deep CNN was inspired by the Alex network [24, 26] used in the championship of ILSVRC and was altered to implement the classification and grading for slit-lamp images. The overall architecture of our CNN, as is shown in Fig 5(a), mainly consists of five convolutional (see Fig 5(b) and 5(c)) and overlapping max pooling layers (see Fig 5(d)) followed by three full-connected layers. Adjacent layers can be connected by edges that are trainable parameters; the inner layer is unconnected. The first seven layers are used to extract multidimensional and high-level features from the raw input image, and the last softmax layer is applied to classification and grading; additionally, we can select the SVM classifier instead of softmax. In this deep CNN, the following crucial technologies are implemented: convolution, overlapping pooling, non-saturating rectified linear units (ReLUs), dropout, data augmentation, and softmax or SVM classifier.

The convolutional layer has two vital merits: local receptive field and shared weights. Each convolution kernel can be seen as a local feature extractor used to identify the relationships between pixels of a raw image so that the effective and appropriate high-level features are extracted to enhance the generalization ability of the CNN model. Furthermore, shared weights can greatly reduce the number of trainable parameters. The result of one convolution operation on $x_{i}^{L-1}$ by $k_{ij}^{L}$ can be represented as Fig 5(b), in which $x_{i}^{L-1}$ stands for the *i*-th input feature map in the *L*-1 layer and $k_{ij}^{L}$ indicates the corresponding learnable kernel. Thus, the convolution output $g_{j}^{L}$ of *L* layer can be re-written as Eq 1, where *M*_*j*_ represents all the input maps involved in the convolution operation.

$$g_{j}^{L}=\sum_{i\in M_{j}}{\text{g}}_{ji}^{L}=\sum_{i\in M_{j}}x_{i}^{L-1}*k_{ij}^{L}$$ (1)

After the convolution operation, the pre-activation $g_{j}^{L}$ combined with the additive bias b enter into the non-saturating nonlinear activation function ReLU to produce the output feature map $x_{j}^{L}$ as is shown in Fig 5(c). This function ReLU [35] is significantly faster than saturating functions such as the sigmoid and hyperbolic tangent. In addition, it can prevent overfitting and improve accuracy. To be more precise, it is the overlapping max pooling layer proposed previously [35] instead of the non-overlapping pooling layer that is investigated and adopted. It aims to achieve spatial invariance and enhance anti-noise capacity by reducing the data dimension of the feature maps. The operation is shown in Fig 5(d): every 3*3 pixels are pooled to one pixel. For example, the 9 pixels surrounded by a red circular area are pooled into P1; then, the blue circular area results in P2 after the pooling kernel moves forward by 2 pixel steps.

It should be clear that the quantity of samples directly affects the depth, accuracy and number of parameters of the CNN model. It is more inclined to be overfitting when few samples are used. Thus, two data augmentation methods (namely, transformed images and horizontal reflections [36]) are adopted to artificially enlarge the slit-lamp dataset. By randomly extracting 224*224 patches from the original image and horizontally flipping them, the training dataset is enlarged by a factor of 2048. By this scheme, the CNN can be trained on slit-lamp images. To further address the overfitting problem, the powerful and efficient technology “dropout” [37] was adopted. Specifically, during the training phase half of the neurons of the hidden layer are selected randomly to be involved whereas during the test phase the output of all neurons are calculated and multiplied by a factor of 0.5. By this technique, co-adaptations of different neurons can be considerably reduced and the performance of the whole network can be enhanced. When the training sample is smaller, the improvement is greater.

In this paper, these is a training set {(*x*^(1)^,*y*^(1)^),…,(*x*^(*m*)^,*y*^(*m*)^)} of m labeled samples in one training phase, where the input features are *x*^(*i*)^∈*R*^*n*^ and the labels are *y*^*(i)*^∈{1,…,*K*}. As the softmax classifier is used in CNN network, the overall loss function *J*(*w*)for a mini-batch training is defined as the Eq 2, which is the cross-entropy cost function adding a weight decay term.

$$J(w)=-\frac{1}{m}\left[\sum_{i=1}^{m}\sum_{j=1}^{k}I\left\{y^{\left(i\right)}=j\right\}\text{log}\frac{e^{w_{j}^{T}x^{\left(i\right)}}}{\sum_{s=1}^{k}e^{w_{s}^{T}x^{\left(i\right)}}}\right]+\frac{\lambda }{2}\sum_{i=1}^{k}\sum_{j=1}^{n}w_{ij}^{2}$$ (2)

where *w*, *m*, *k* and *n* represent the trainable weights, the mini-batch size, the number of categories and the number of input nodes of softmax function respectively. The *I*{·} is the indicator function (*I*{*a true statement*} = 1, and *I*{*a false statement*} = 0), and $\frac{\lambda }{2}\sum_{i=1}^{k}\sum_{j=1}^{n}w_{ij}^{2}$ is a weight decay term which penalizes large values of the weights. To achieve the minimization of the loss function, the stochastic gradient descent (SGD) with mini-batch [38], a very simple and efficient method, is employed to optimize the parameters of the CNN (Details on the network parameters can be found elsewhere [24, 26]). To apply the SGD with mini-batch algorithm, we also need solve the derivative of the loss function *J*(*w*) as the Eq 3, and the weights of the other layers can be obtained by an application of the back-propagation chain rule.

$$\nabla_{w_{j}}J(w)=-\frac{1}{m}\sum_{i=1}^{m}\left[x^{\left(i\right)}(I\{y^{\left(i\right)}=j\}-p(y^{\left(i\right)}=j|x^{\left(i\right)};w))\right]+\lambda w_{j}$$ (3)

By minimizing *J*(*w*)with respect to w, we will obtain the optimal weights w* as Eq 4.

$$w^{*}=\text{arg }\underset{w}{\text{min}}J(w)$$ (4)

In this study, we set the size of the stochastic mini-batch to one eighth of the entire training samples, accelerating parameter training convergence. The learning rate was initialized at 0.01 and successively decreased to one tenth of the original per 500 iterations, and the maximum number of iterations was 2000. These parameters are ideal for our datasets in preventing the CNN model from divergence. The CNN was trained using an NVIDIA TITIAN X GPU in Ubuntu 14.04 based on the Caffe toolbox [39].

## Results and discussion

### Dataset

The slit-lamp image datasets were obtained from the Zhongshan Ophthalmic Center in Sun Yat-sen University, the leading eye hospital in China, which has collected comprehensive electronic medical records (EMR) for ophthalmic diseases. The sharing of datasets for pediatric cataracts and other rare diseases was proposed by Haotian Lin [29] in Science in September 2015. These datasets and studies provided an ideal basis for our research. To avoid duplicates and ensure the representative and accurate images, each image was discussed and confirmed by three experienced ophthalmologists to determine whether it was correctly enrolled and categorized. There is no special demand for slit-lamp images and their pixels. The slit-lamp images that contain the valuable lens are eligible for training and testing. For a very few slit-lamp images, very blurry, their meaningful clinical information could not be identified by three ophthalmologists, these slit-lamp images were excluded after experts’ discussion. The excluded images were very rare cases in our experiments. Therefore, the applicability and robustness of our training model is quite extensive. The final dataset included 886 images, of which 476 depicted normal tissue and 410 depicted pediatric cataracts of various degrees of severity. Using three degrees of morphology, all samples of pediatric cataracts were further graded: limited (172) and extensive (238) for area, dense (231) and transparent (179) for density, and central (260) and peripheral (150) for location.

### Evaluation metrics

To evaluate the performance of our proposed framework compared with traditional methods, qualitative and quantitative measures are employed. Based on these two aspects, we can claim that the high-level features that are extracted from slit-lamp images using CNN are effective and discriminatory and that both softmax and SVM are excellent classifiers.

The quantitative measures we choose are accuracy (ACC), sensitivity (SEN), specificity (SPC), receiver operation characteristic (ROC) curves, and loss function (*J*(*w*)), which are generic evaluation indicators for classification methods. To analyze the reliability and generalization ability of the CNN method, the data sets are divided into four groups with approximately equal size, and the well-known k-fold cross-validation (CV) technique [40] is applied to obtain the mean and standard deviation for ACC, SEN and SPC.

Accuracy=(TP+TN)/(TP+FN+TN+FP)

Sensitivity=TP/(TP+FN)

Specificity=TN/(TN+FP)

where TP, FP, TN and FN stand for the number of true positives, false positives, true negatives and false negatives in the detection results respectively. Similar to other medical problems, positive implies a pediatric cataract in classification and a relatively serious condition in grading whereas a negative sample is the opposite.

The mean ACC with standard deviation is the principal indicator: the higher the value of ACC, the better performance is demonstrated by the classifier. The sensitivity and specificity constitute a pair of indicators; a higher sensitivity implies that pediatric cataract patients are detected more easily, whereas a higher specificity indicates that a normal person can be identified more precisely. The ROC is another vital objective evaluation in the task of image classification, which is depicted by true positive rate (sensitivity) and false positive rate (1-specificity); the larger area under the ROC curve, the better is the classification performance. In addition, the loss value of *J*(*w*) decreases gradually with further iteration, which indicates that the CNN is convergent.

The t-distributed stochastic neighbor embedding (t-SNE) method proposed by Hinton has been proven to be an effectively qualitative indicator for intuitively characterizing the performance of feature extraction. The CNN features can be visualized by t-SNE, which maps the feature space from high-dimension to low-dimension. In this study, we choose two-dimensional space as the mapping space because a more linearly separable two-dimensional map ensures better feature extraction performance.

### Qualitative results

**Fig 6:**
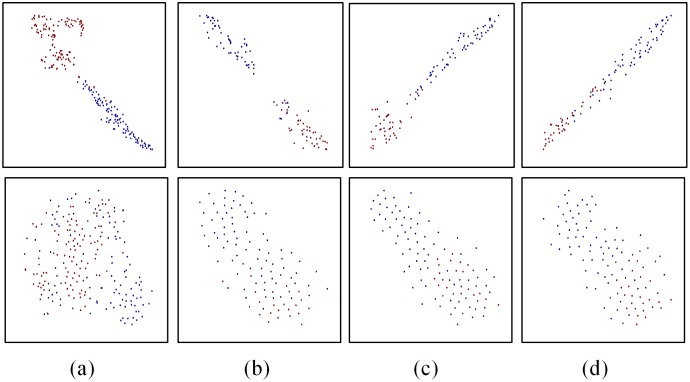
The t-SNE maps of CNN and traditional features. From (a) to (d) are the classification and three-degree grading: area, density, location, in which the upper images are maps of FC7 features in CNN network and the lower images are typical traditional features wavelet transformation. And the blue and red dots represent samples of two different categories. (t-SNE: t-distributed stochastic neighbor embedding; CNN: convolutional neural network; FC7: the seventh fully-connected layer).

First, we investigate qualitatively whether the extracted features from CNN network are discriminative, and we choose the representative wavelet transformation features for comparison. As is shown in Fig 6, the t-SNE method is used to generate visualizing maps (classification and grading) of the seventh fully-connected (FC7) layer features in our CNN and wavelet transformation features. From all maps, we can obviously notice that different types of samples are almost separated linearly in two-dimensional space using CNN features. On the contrary, it is very difficult to separate them using wavelet transformation features. This result appears to suggest that the deep features extracted using CNN are meaningful and discriminative and can be used to identify different types of samples easily.

### Quantitative results

To further explore the effect of the deep features extracted by our approach, we conducted comparative experiments and quantitatively analyzed the statistical results in terms of accuracy, sensitivity and specificity using their mean and standard deviation.

In our previous study, a number of feature extractors and classifiers were investigated to analyze their performance and efficiency for characterizing slit-lamp images. The features extraction methods primarily involve the color feature, texture feature, wavelet transformation (WT), local binary pattern (LBP), scale-invariant feature transform (SIFT), whereas the classifiers include the SVM, extreme learning machine (ELM), sparse representation (SP), and k-nearest neighbor (KNN). The color and texture features (COTE) are employed to realize a combination of features. In this paper, we compare only the representative feature extraction methods—WT, LBP, SIFT and COTE, which achieved superior performance in our previous research. We chose the comparison classifier SVM, which has performed well in many studies and was implemented using the LIBSVM [41] toolbox. In addition, many experiments of detailed comparison were conducted to select optimal parameters for the radial basis function (RBF) of the SVM. Experimental results indicate that the performance of the linear kernel with default parameters is almost equal to the RBF kernel with optimal parameters in the SVM classifier.

As is shown in Table 1, we compute the above quantitative measures with mean and standard deviation on 4-fold cross validation experiments; it can be observed that the deep learning CNN model achieves remarkable performance and desirable recognition rates of classification and grading (as shown in bold), which are far beyond the traditional methods in terms of ACC, SPE and SNE. With the exception of our approach, the WT features give relatively satisfactory performance in terms of classification, area and density grading, whereas the SIFT method is suitable for location grading. Each statistical result consists of a mean and standard deviation, and the number in the parentheses is the standard deviation. The bolded and underlined numbers represent the first and second performance for a specific quantitative measure.

**Table 1:** Quantitative evaluation for pediatric cataract classification and grading results with different CAD models.

	Metrics	WT	LBP	SIFT	COTE	CNN
Classification	ACC (%)	88.26 (0.02) ^§^	85.10 (0.02)	77.76 (0.01)	71.12 (0.13)	**97.07 (0.01)**
	SPC (%)	95.38 (0.03)	87.82 (0.01)	88.03 (0.04)	74.58 (0.39)	**97.28 (0.01)**
	SEN (%)	80.00 (0.06)	81.95 (0.05)	65.85 (0.03)	67.06 (0.19)	**96.83 (0.02)**
Area grading	ACC (%)	78.29 (0.03)	70.72 (0.06)	76.09 (0.04)	74.17 (0.08)	**89.02 (0.01)**
	SPC (%)	76.74 (0.12)	56.40 (0.11)	81.98 (0.04)	59.88 (0.36)	**86.63 (0.06)**
	SEN (%)	79.41(0.06)	81.07 (0.04)	71.81(0.09)	84.34(0.15)	**90.75(0.04)**
Density grading	ACC (%)	83.39 (0.03)	71.00 (0.04)	77.32 (0.02)	82.96 (0.05)	**92.68 (0.01)**
	SPC (%)	82.69 (0.03)	62.64 (0.09)	83.83 (0.04)	79.99 (0.13)	**91.05 (0.02)**
	SEN (%)	83.95 (0.07)	77.49 (0.01)	72.29 (0.05)	85.28 (0.07)	**93.94 (0.02)**
Location grading	ACC (%)	78.77 (0.03)	76.09 (0.04)	80.73 (0.01)	67.83 (0.07)	**89.28 (0.03)**
	SPC (%)	71.32 (0.08)	56.65 (0.06)	74.77 (0.09)	52.83 (0.37)	**82.70 (0.06)**
	SEN (%)	83.08 (0.04)	87.31 (0.04)	84.23 (0.05)	76.54 (0.23)	**93.08 (0.04)**

Footnotes: ACC (accuracy); SPC (specificity); SEN (sensitivity); WT (wavelet transformation); LBP (local binary pattern); SIFT (scale-invariant feature transform); COTE (color and texture features); CNN (convolutional neural network);

^§^Mean (Standard Deviation).

**Fig 7:**
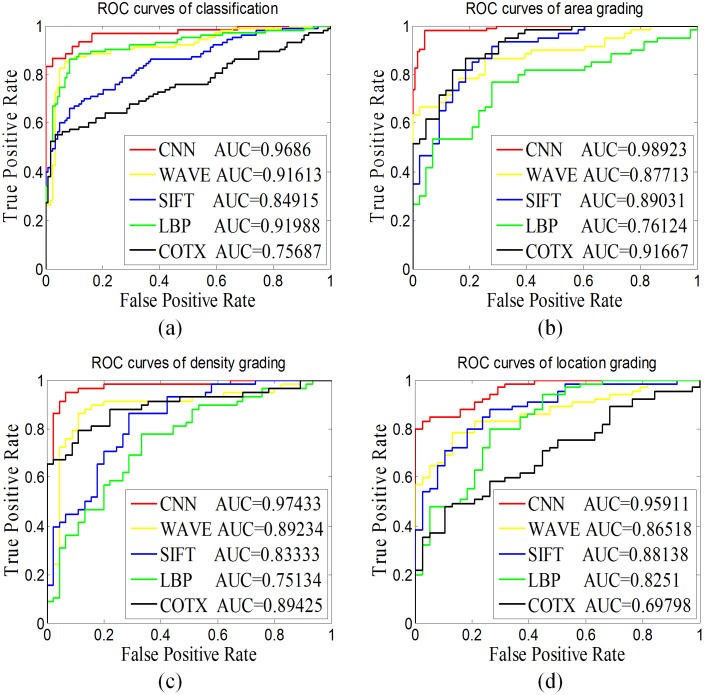
The ROC curve and AUC value for pediatric cataract. This figure contains the ROC curve and AUC value of **(a)** classification and three-degree grading **(b)** area, **(c)** density, and **(d)** location with different CAD models. (ROC: receiver operating characteristics curve; AUC: area under the curve; CAD: computer aided diagnosis; CNN: convolutional neural network; WAVE: wavelet transformation; LBP: local binary pattern; SIFT: scale-invariant feature transform; COTE: color and texture features).

Furthermore, we chose the ROC curve and AUC (area under the curve) value to investigate the reliability and generalization ability of our proposed CNN model to compare with traditional methods shown in Fig 7. To obtain a more accurate comparison result, we ensured that the same set of training and testing datasets were used for every method. We conducted experiments of detailed comparison and obtained the ROC curves of five methods depicted as classification (a) and grading area (b), density (c) and location (d) in Fig 7. The CNN achieves better performance than the other hand-crafted features. The AUC values indicate that our proposed CNN is the superior method as measured by classification (0.9686) and the three-degree grading area (0.98923), density (0.97433) and location (0.95911).

**Fig 8:**
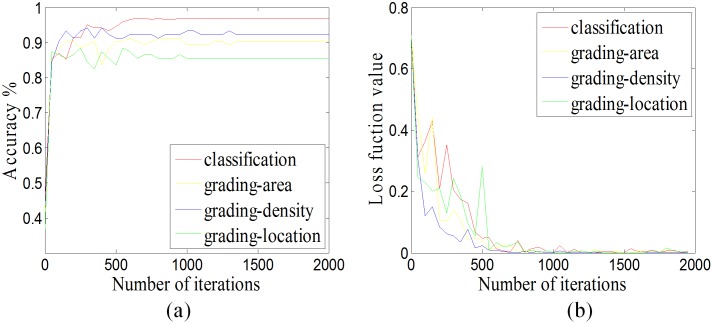
The convergence analysis. **(a)** The accuracy curve in respect to iterations. **(b)** The loss function value curve in respect to iterations.

To verify the convergence and effectiveness of our CNN for pediatric cataracts in classification and grading, in the process of training, we performed one test every 50 iterations to obtain accuracy and loss function values, which were used to characterize the change in ACC and loss function value with iterations. As is shown in Fig 8, the ACC rapidly increases with iteration number, whereas the loss function value decreases; and eventually they stabilize. These results suggest that the CNN model for slit-lamp images is convergent and effective.

**Fig 9:**
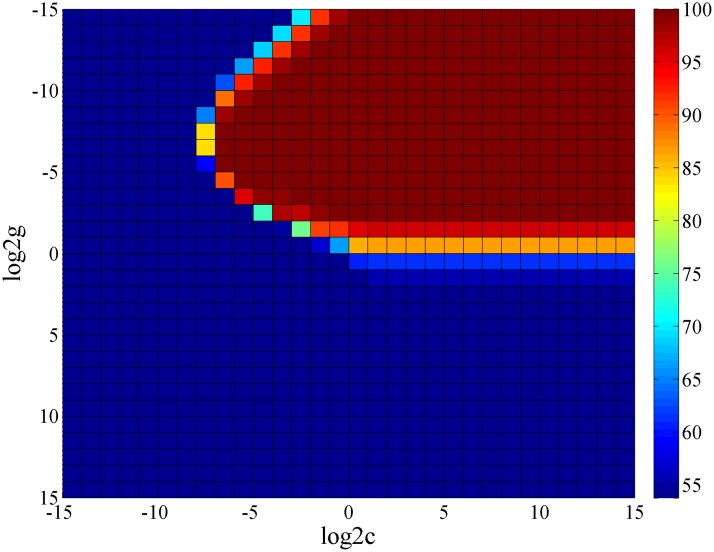
Heat map of the performance of RBF kernels with different values of c and gamma. Different colors represent different levels of accuracy, and the accuracy increases from blue to red. (RBF: radial basis function; c: cost parameter of RBF; g: gamma parameter of RBF).

Furthermore, the classifiers for classification and grading in Fig 2 are not restricted to softmax; the CNN is primarily used to obtain universal high-level features from raw slit-lamp images, whereas the softmax or other classifiers can be chosen. As is shown in Fig 5, we selected softmax, which was the native classifier in the CNN for investigating CAD for slit-lamp images. We subsequently attempted to improve the CNN by proposing a new scheme for pediatric cataracts, which combined the high-level features of the FC7 layer in the CNN with an SVM classifier. Our goal is to improve the recognition rate using the superior performance of the SVM classifier. Generally, in all kernel function of SVM, the RBF was no worse than the linear approach, so that we applied the RBF kernel of SVM for contrastive analysis and used the grid method to select the optimal c (cost) and g (gamma) parameters, which determined the performance of the RBF kernel. For example, we obtained the heat map of classification performance with different parameters c and gamma (Fig 9). The optimization accuracy was 96.85% (log2c = -1, log2g = -8), which is equivalent to the linear kernel. However, the time consumption of RBF is greater than the linear kernel; therefore, a trade-off exists between efficiency and effectiveness when the basic linear SVM classifier is used.

Using the same approach, we obtained three sets of comparison results shown in Table 2. From top to bottom, there are the CNN classifier (softmax), the SVM classifier (linear kernel function) combined with the FC7 features of CNN, and the SVM classifier (RBF) combined with the FC7 features of CNN respectively. The ACC of the SVM classifier is slightly better than softmax with regards to density and location grading. For classification and area grading, the ACC of SVM is equivalent to softmax. The performance of SVM and softmax are almost equivalent regarding the deep features of CNN, and the excellent experiment results verify that the features extracted using CNN are effective and representative.

**Table 2:** Comparison of SVM and softmax classifier with FC7 high-level features extracted by the CNN.

Method	Classification	Area grading	Density grading	Location grading
CNN	**96.85%**	**90.29%**	92.23%	88.35%
CNN_FC7 (Linear SVM)*	**96.85%**	**90.29%**	**93.20%**	89.32%
CNN_FC7 (RBF SVM)^§^	**96.85%**	**90.29%**	92.23%	**90.29%**
	log2c = -1; log2g = -8	log2c = -6; log2g = -10	log2c = -6; log2g = -8	log2c = -5; log2g = -8

Footnotes: CNN (convolutional neural network); SVM (support vector machine); CNN_FC7 (the seventh fully-connected layer features of CNN); RBF (radial basis function);

*(linear kernel function SVM classifier);

^§^(RBF SVM classifier); c (cost parameter of RBF); g (gamma parameter of RBF).

### Web-based software

The above abundant comparative experiments and detailed analysis demonstrate that the CNN model achieves remarkable performance and desirable recognition rates. The website of our system provides detailed instructions for our software to be easily operated. Anyone can access our software using the website: https://www.cc-cruiser.com/slit-lamp. This software could characterize pediatric cataracts using automatic localization of lens area and the CNN method, and this software has been applied in Zhongshan Ophthalmic Center, Sun Yat-Sen University. This CAD system can not only assist proposals of treatment for ophthalmologists but also enable high quality medical care and individualized treatment for the patients in developing areas where sophisticated medical devices and well-trained doctors are scarce. In addition, this software can be used in teaching activities for junior students of ophthalmology. This software supports prediction of multiple images simultaneously. Furthermore, with the extensive use of this software, we will collect an extensive range of slit-lamp images to enhance the accuracy of the CNN model with larger data sets.

## Conclusions and future work

In this paper, we presented a deep-feature-based CAD framework for classifying and grading slit-lamp images. First, we analyzed the complexity of slit-lamp images for pediatric cataracts and proposed three-degree grading in terms of morphology. Then, the ROI of the lens was identified with the adoption of twice-applied Candy detection and Hough transform and then it entered into the CNN to investigate the slit-lamp image. According the results of quantitative measures, the overall performance of our proposed CNN method is significantly better than the representative customized feature methods. Qualitative assessment also indicates that the high-level features extracted from the CNN is discriminative, and we combined these high-level features with an SVM classifier to improve the results. As a result, this work addressed significant needs in pediatric cataract research and may shed a light on other ocular images. Finally, we developed automatic diagnosis software to realize clinical application for ophthalmologists and patients.

Due to the basic requirements for high accuracy and reliability in clinical diagnosis, our future study will analyze the characteristics of features that have been extracted from deep learning CNN. We will combine other features to improve the performance of CAD. To achieve this goal, we plan to deploy multi kernel learning and ensemble learning for slit-lamp images. After a period of clinical application, we hope to take full advantage of the deep learning model to enhance the reliability of the diagnosis system with large amounts of accumulated datasets. We also plan to apply the proposed method to other biomedical images and assess its performance and robustness using multiple datasets.
